# Attentional modulation of desensitization to odor

**DOI:** 10.3758/s13414-018-1539-2

**Published:** 2018-05-22

**Authors:** Nicholas Fallon, Timo Giesbrecht, Andrej Stancak

**Affiliations:** 1 Department of Psychological Sciences, Institute of Psychology, Health, and Society, University of Liverpool, Eleanor Rathbone Building, Bedford Street South, Liverpool, L69 7ZA UK; 2 Unilever Research & Development, Port Sunlight, UK

**Keywords:** Olfaction, Adaptation and aftereffects, Cognitive and attentional control

## Abstract

Subjective and behavioral responsiveness to odor diminishes during prolonged exposure. The precise mechanisms underlying olfactory desensitization are not fully understood, but previous studies indicate that the phenomenon may be modulated by central-cognitive processes. The present study investigated the effect of attention on perceived intensity during exposure to a pleasant odor. A within-subjects design was utilized with 19 participants attending 2 sessions. During each session, participants continuously rated their perceived intensity of a 10-minute exposure to a pleasant fragrance administered using an olfactometer. An auditory oddball task was implemented to manipulate the focus of attention in each session. Participants were instructed to either direct their attention toward the sounds, but still to rate odor, or to focus entirely on rating the odor. Analysis revealed three 50-second time windows with significantly lower mean intensity ratings during the distraction condition. Curve fitting of the data disclosed a linear function of desensitization in the focused attention condition compared with an exponential decay function during distraction condition, indicating an increased rate of initial desensitization when attention is distracted away from the odor. In the focused-attention condition, perceived intensity demonstrated a regular pattern of odor sensitivity occurring at approximately 1−2 minutes intervals following initial desensitization. Spectral analysis of low-frequency oscillations confirmed the presence of augmented spectral power in this frequency range during focused relative to distracted conditions. The findings demonstrate for the first time modulation of odor desensitization specifically by attentional factors, exemplifying the relevance of top-down control for ongoing perception of odor.

**Electronic supplementary material:**

The online version of this article (10.3758/s13414-018-1539-2) contains supplementary material, which is available to authorized users.

Psychophysical studies have shown that perceived odor intensity diminishes during the course of prolonged or repetitive olfactory stimuli (Berglund, 1974; Cain, 1969; Ekman, Berglund, Berglund, & Lindvall, 1967). It is generally accepted that a reduction of perceived odor intensity occurs concurrently at both peripheral and central levels. Peripheral reductions in responsiveness that occur at the level of olfactory receptor neurons (Hummel, Knecht, & Kobal, 1996; Kurahashi & Menini, 1997) are often referred to as adaptation, whereas central-cognitive processes (such as changes in brain activity or reduced behavioral responsiveness) may be termed habituation (Dalton, 2000; Thompson & Spencer, 1966). Eletro-olfactogram recordings directly from olfactory receptors demonstrated that behavioral desensitization is independent from adaptation in the periphery (Hummel et al., 1996), and functional magnetic resonance imaging (fMRI) studies revealed blood-oxygen-level-dependent (BOLD) signal changes associated with desensitization to odor stimuli that were predominantly encoded in primary olfactory cortices (Poellinger et al., 2001). However, despite this functional and semantic demarcation, it is clear that central and peripheral processes occur in parallel and share a complex relationship (Pellegrino, Sinding, de Wijk, & Hummel, 2017), and it is their cumulative effects that determine subjective changes in perception (Dalton, 2000). This holistic decrease in perceived odor intensity can be referred to as olfactory desensitization (Stuck, Fadel, Hummel, & Sommer, 2014). Further investigation of peripheral and central-cognitive processes in isolation is critical to improving our understanding of the mechanisms underlying olfactory desensitization.

One way to investigate central-cognitive aspects of desensitization in isolation is via novel experimental paradigms that target central processing. Attention can be directed toward olfaction in a similar fashion to other sensory modalities (Keller, 2011), and the composition of perceived “odor objects” can be influenced by top-down processes (Wilson & Sullivan, 2011). Previous research has demonstrated that allocation of attention toward stimuli reduces response times for odor perception (Spence, Kettenmann, Kobal, & McGlone, 2000, 2001; Spence, McGlone, Kettenmann, & Kobal, 2001) and also modulates perceptions of odor intensity (Rolls, Grabenhorst, Margot, da Silva, & Velazco, 2008; Spence, McGlone, et al., 2001). Electroencephalographic (EEG) studies have shown that focused attention increases olfactory event-related potentials (Geisler & Murphy, 2000; Krauel, Pause, Sojka, Schott, & Ferstl, 1998; Masago, Shimomura, Iwanaga, & Katsuura, 2001; Pause, Sojka, & Ferstl, 1997), and fMRI studies report variations in hemodynamic brain responses when attention is focused toward an odor (Plailly, Howard, Gitelman, & Gottfried, 2008; Sabri, Radnovich, Li, & Kareken, 2005; Veldhuizen & Small, 2011; Zelano et al., 2005) or toward a particular quality of an odor (Rolls et al., 2008). Moreover, research indicates that the process of olfactory desensitization may be influenced by varying top-down factors such as odor hedonics (Jacob, Fraser, Wang, Walker, & O’Connor, 2003), prior experience (Smeets & Dalton, 2002; Wysocki, Dalton, Brody, & Lawley, 1997), or cognitive manipulations of odor salience (Dalton, 1996; Dalton, Wysocki, Brody, & Lawley, 1997; Kobayashi et al., 2008; Smeets & Dalton, 2005).

Despite the previous research reviewed above, to our knowledge no study has investigated how focus of attention itself may affect the process of olfactory desensitization. In the present work, we investigated the effects of attention on perceived intensity of a pleasant odor that was presented for a 10 minute period to induce desensitization. The stimulus was presented continuously and controlled using an olfactometer with constant odor concentration and flow rate to minimize variation in the periphery. A within-subjects design was utilized, and an auditory oddball task was employed to manipulate focus of attention during the period of olfactory desensitization.

## Materials and methods

### Participants

Nineteen participants (seven males) ages 26.8 ± 3.0 years (*M* ± *SD*) took part in the study. Participants were recruited through campus advertisement at the University of Liverpool. Informed consent was obtained from all participants in accordance with the Declaration of Helsinki, and the study was approved by the University Research Ethics Committee at the University of Liverpool. Participants between 18 and 35 years of age were considered for participation, and volunteers taking regular medication or those suffering from respiratory, neurological, or olfactory disease or disorders were excluded. The number of participants was based on previous psychophysical studies of prolonged odor exposures (Stuck et al., 2014) or investigations of effects of attention on olfaction (Spence, McGlone, et al., 2001).

Eligibility and sense of smell was assessed prior to the experiment using the identification test from the Sniffin’ Sticks odor test battery (Hummel, Sekinger, Wolf, Pauli, & Kobal, 1997). Participants were asked to identify 12 odors from four visually presented options, and a minimum score of 9 correct probes was required for inclusion in the study. Participants were scheduled to attend two experimental sessions 1 week apart: In one session they focused on the odor (focused attention condition), and in another their focus was distracted away from the olfactory stimulus by means of an auditory oddball task (distraction condition). The order of conditions was balanced across participants. All volunteers were compensated for time and travel expenses following completion of the study.

### Odor stimuli

The odor utilized for the study was Snow Queen 10, a pleasant floral, green fragrance with minimal trigeminal activity according to component ingredients and profile (UR224459/00, Givaudin Ltd., Switzerland). For the purpose of the experiment a 0.1% (v/v) dilution of fragrance in propylene glycol (1,2-Propanediol 99%, Sigma-Aldrich Co., USA) was prepared. For the clean-air control stimulus, pure propylene glycol solution was used. The olfactometer utilized was a custom-made flow olfactometer, with eight individual valves allowing for variable flow rates and a carbon filtered air intake (OL-2, Dancer Design Ltd., UK). The olfactometer delivered odors via two polytetrafluoroethylene tubes of 2-mm diameter ending approximately 2 cm below the nostrils. The flow rate was set to 2.2 liters/minute at the beginning of each experimental session and exhibited minimal variation (<0.1 l/min) throughout the course of exposure to ensure odor concentration was maintained. Delivery of the odor was preceded and succeeded by clean air at a matching flow rate to avoid sudden increases in airflow associated with presentation of an odor. Before and after each experiment, the ambient air in the chamber was cleansed of residual odors using a carbon filtered Blueair 203 Heppasilent Particle Filter system (Blueair AB, Sweden).

### Procedure

Participants were tested in a sound-attenuated chamber, and clean, carbon-filtered air was pumped from outside the room, through the olfactometer, to the nosepiece. A PneumoTrace II Piezo-electric transducer was fitted around the torso at the level of the epigastrium to record respiratory movements (AD Instruments Pty Ltd., Australia). Participants were seated approximately 0.7 m from a 19-inch monitor. Prior to the experiment, each participant rated odor stimuli for intensity, pleasantness, and familiarity using visual analogue scales (VAS). For intensity ratings, the VAS anchors were *not perceivable* to *extremely Intense*; for pleasantness, *extremely unpleasant* to *extremely pleasant*; and for familiarity, *extremely unfamiliar* to *extremely familiar*.

After initial evaluations of the odor, participants were provided with a response meter (AD Instruments Pty Ltd., Australia) with a slide-bar control that allowed them to continuously quantify their response to the stimulus. They were instructed that the slide bar was for continuous rating of the intensity of the odor and underwent brief training for suitable use. The anchors on the slide bar ranged from *not perceivable* to *extremely intense*. All participants were instructed that odors may or may not be presented at any time during the session and that they should try to be as fast and accurate as possible with their continuous ratings.

During all experimental sessions, the odor presentation was accompanied by an auditory oddball task. Auditory beep tones (total = 180) were presented at 60-dB sound pressure level using stereophonic speakers positioned 0.8 m from the subject. Stimuli were presented at pseudorandom intervals (range 3−5 s, mean interstimulus interval 4 s), and thirty six tones (20%, interspersed randomly throughout the session) were presented at a noticeably higher pitch (800 Hz) to the majority (500 Hz). In the distraction condition, participants were instructed to maintain a running count of the total number of “odd” beeps (those with a higher pitch). They were informed that their final count would be required at the completion of the session, but that continuous rating of odor intensity using the response meter was still a necessary task. In the focused-attention condition, participants were informed that they would hear a series of tones during the experiment, but that they should ignore them and focus entirely on rating any odor that they may detect. The order of conditions was counterbalanced across sessions.

In both conditions, the experimental paradigm encompassed a 60-s presentation of clean air, followed by a 600-s presentation of the pleasant odor, followed by a 60-s presentation of clean air (flow rate 2.2 l/min). During the experiment, the monitor displayed basic instructions, reminding participants to relax and breathe normally through their nose, to rate the intensity of any odor they may perceive, and to either *IGNORE the beeps* or *FOCUS on counting the odd beeps*. Auditory and odor stimuli were administered using Cogent 2000 software in MATLAB 7.13 (The MathWorks Inc., Natick, MA, USA).

### Data acquisition

Data were recorded using an AD Instruments PowerLab 8/35 system (AD Instruments Ltd., Australia) and LabChart 7.3 acquisition software (AD Instruments Ltd., Australia) at a sampling rate of 1000 Hz.

### Analysis

Raw data for subjective ratings of intensity were exported to MATLAB 7.13 (The MathWorks Inc., Natick, MA, USA) and resampled to 1 Hz, resulting in 720 samples. The maximum perceived odor intensity during the first 10 seconds of odor presentation was exported for each individual in both conditions, and a within-subjects *t* test was utilized to compare maximum perceived odor intensity between conditions. A within-subjects *t* test was performed to compare mean intensity ratings at each of the 720 samples in the experiment, and permutation analysis (2,000 permutations) was employed to correct for the large number of tests required (Maris & Oostenveld, 2007). Time periods exhibiting consistent differences in mean intensity ratings were identified, and the average intensity rating during each epoch was determined. A within-subjects *t* test, or Wilcoxon signed-rank test (depending on distribution of data), was performed to compare mean intensity ratings across both conditions in each of these time periods of interest.

### Curve-fitting analysis

To further compare the mean rate of habituation during distraction and focused attention conditions, mean data from the time point of the maximum perceived odor intensity until 120 seconds later (representing the period of desensitization from maximum to minimum intensity ratings) was determined for distraction and focused attention data sets. The curve fitting toolbox in MATLAB (The MathWorks Inc., Natick, MA, USA), was employed to identify the mathematical function that best fitted the group data set for each condition.

### Spectral analysis

To evaluate possible low-frequency oscillatory changes in perceived odor intensity during focused attention and distraction conditions, perceived intensity data for the period 120 s to 660 s following onset of odor was exported for each subject. This time window covered the period following the rapid initial desensitization (which was investigated using the previously described curve-analysis method). The relative power spectral density was estimated using Welch’s averaged modified periodogram method. This yielded a power spectral density ranging from 0 to 0.5 Hz (129 components) for each subject and condition. A within-subjects *t* test was utilized to compare mean intensity ratings at each of 129 frequency components, and permutation analysis (2,000 permutations) employed to correct for multiple tests.

### Respiratory data analysis

The respiratory movement signals were resampled to 10 Hz, and inspiratory peaks occurring in each 10-min recording were identified. Respiration rate (cycles/minute) was evaluated for time periods showing significant differences in intensity ratings between conditions, and mean respiration rates during these periods were compared between conditions using a within-subjects *t* test. In addition, we calculated mean respiration rates for the period of exposure for each subject using a 60-s moving window average (which necessitated the removal of 30 s of data at the beginning and end of time series). Pearson’s correlation coefficients for individual respiration rate and intensity ratings were calculated for each condition. One-sample *t* tests were utilized to investigate whether individual correlations between respiration rate and odor perception across the group significantly differed from the null, and a paired-samples *t* test was utilized to evaluate differences between conditions.

## Results

### Pretest VAS odor ratings

Within-subjects *t* tests revealed no significant differences between VAS scores for perceived odor intensities prior to the focused attention (5.29 ± 1.66, *M* ± *SD*), and distraction (4.73 ± 1.71) conditions; *t*(18) = 1.77, *p* = .09, or for perceived pleasantness in focused attention (6.81 ± 0.92) and distraction (6.98 ± 1.01) conditions; *t*(18) = −0.51, *p* = .62.

### In-test intensity ratings

**Fig. 1:**
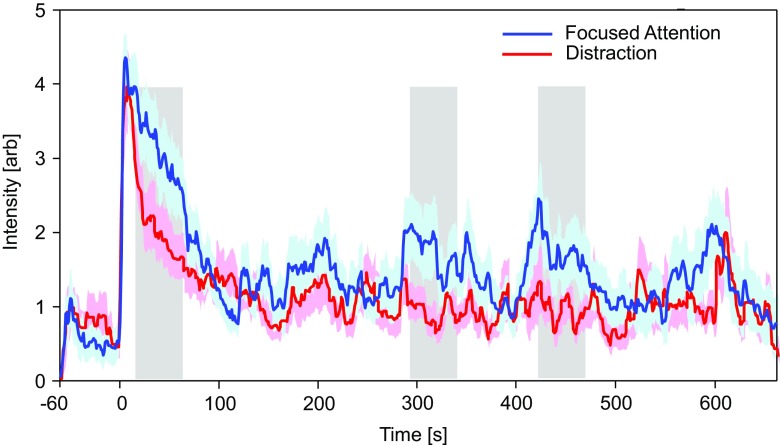
Mean perceived intensity ratings and desensitization curves. Blue indicates mean intensity ratings (arbitrary units) with shading to indicate standard error distribution for all 720 timepoints in the focused attention condition; red indicates the distraction condition. Gray rectangles indicate time periods when mean intensity ratings differ significantly between conditions. (Color figure online)

Mean continuous intensity ratings for both conditions (see Fig. 1) exhibit a steep incline immediately following odor onset and achieve maximal perception within 10 s. This was followed by a gradual decline in perceived intensity which lasted approximately 120 s to minimal perception. The maximum odor intensity perceived during the first 10 seconds of odor presentation was determined for each subject in both conditions. A within-subjects *t* test revealed no significant difference between maximum perceived odor intensities for the focused attention (4.85 ± 1.48) and distraction conditions (4.32 ± 2.11); *t*(18) = 1.13, *p* = .22. The continuous intensity ratings for each participant in both conditions are illustrated in Supplementary Fig. S1.

Permutation analysis identified three time periods when mean intensity ratings differed significantly between conditions (see Fig. 1, gray rectangles). The first significant difference between focused and distraction conditions occurred during the initial period of pronounced desensitization. Later on in the exposure, recurrent phases of significantly increased odor-intensity perception in ratings were evident in the focused-attention condition relative to the distraction condition. The pattern of recurrent-odor perception in the focused-attention condition displayed a quasi-oscillatory profile, which was dominated by low-frequency oscillations lasting in the region of 100 s.

For each time window highlighted by permutation analyses, mean intensity ratings were exported, and a comparison test was performed. At 15 to 65 s following onset of odor stimuli, a significant difference was seen between mean intensity ratings for the focused attention (3.12 ± 1.83, *M* ± *SD*) and distraction conditions (2.02 ± 1.49); *t*(18) = 2.28, *p* = .035. Between 290 and 340 s following onset of odor stimuli, a Wilcoxon signed-rank test showed that intensity ratings for the focused attention condition were significantly greater than during the distraction condition; *z* = −1.97, *p* = .049. Likewise, between 420 and 470 s following onset of odor stimuli, mean intensity ratings for the focused-attention condition were greater than those during the distraction condition; *z* = −2.01, *p* = .044.

### Curve-fitting analysis of initial desensitization

**Fig. 2:**
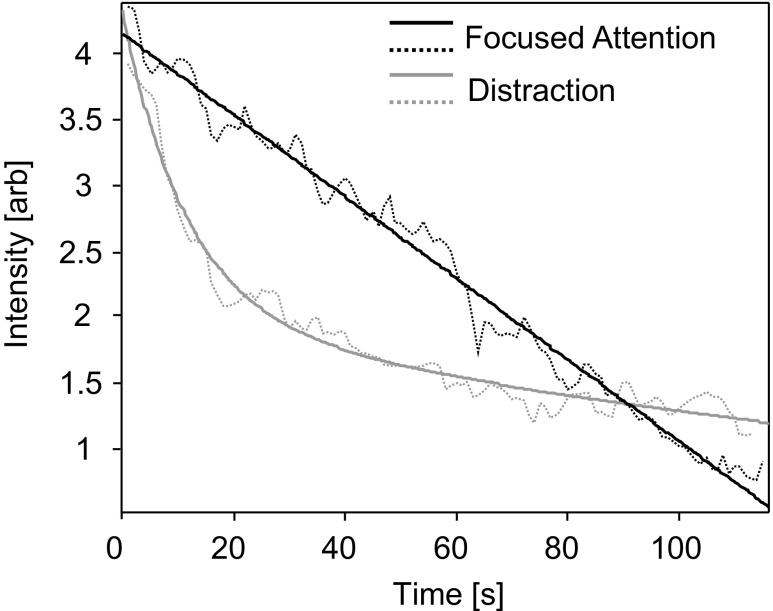
Desensitization data and curve functions of best fit. Black dashed and solid lines show mean intensity ratings (arbitrary units) and curve fitting, respectively, for 120 seconds following maximum perceived intensity in the focused attention condition; gray solid and dashed lines represent data and curve fitting for the distraction condition. Note the divergence between curves in the period 10–80 seconds following maximum perceived odor intensity.

Mean perceived intensity data from the time point of the maximum perceived odor intensity until 120 seconds later (minimal odor perception) was exported for both conditions. The curve function that best explained the data (highest adjusted *R*^2^ value, lowest sum of squared error value) was fitted for each condition (see Fig. 2) in the MATLAB curve-fitting toolbox. A linear polynomial function best described the curve in the focused-attention condition (adjusted *R*^2^ = 0.98, *SSE* = 1.87), whereas a second-order exponential decay function demonstrated the best fit in the distraction condition (adjusted *R*^2^ = 0.97, *SSE* = 1.31). The greatest difference in curve functions covers a similar time period to the first time window, which showed a significant difference in mean intensity ratings. This finding suggests that the steeper exponential rate of desensitization during the distraction condition may account for reduced intensity ratings seen during the time period from 15 to 65 seconds following onset of odor presentation.

### Recurrent perceived odor intensity

**Fig. 3:**
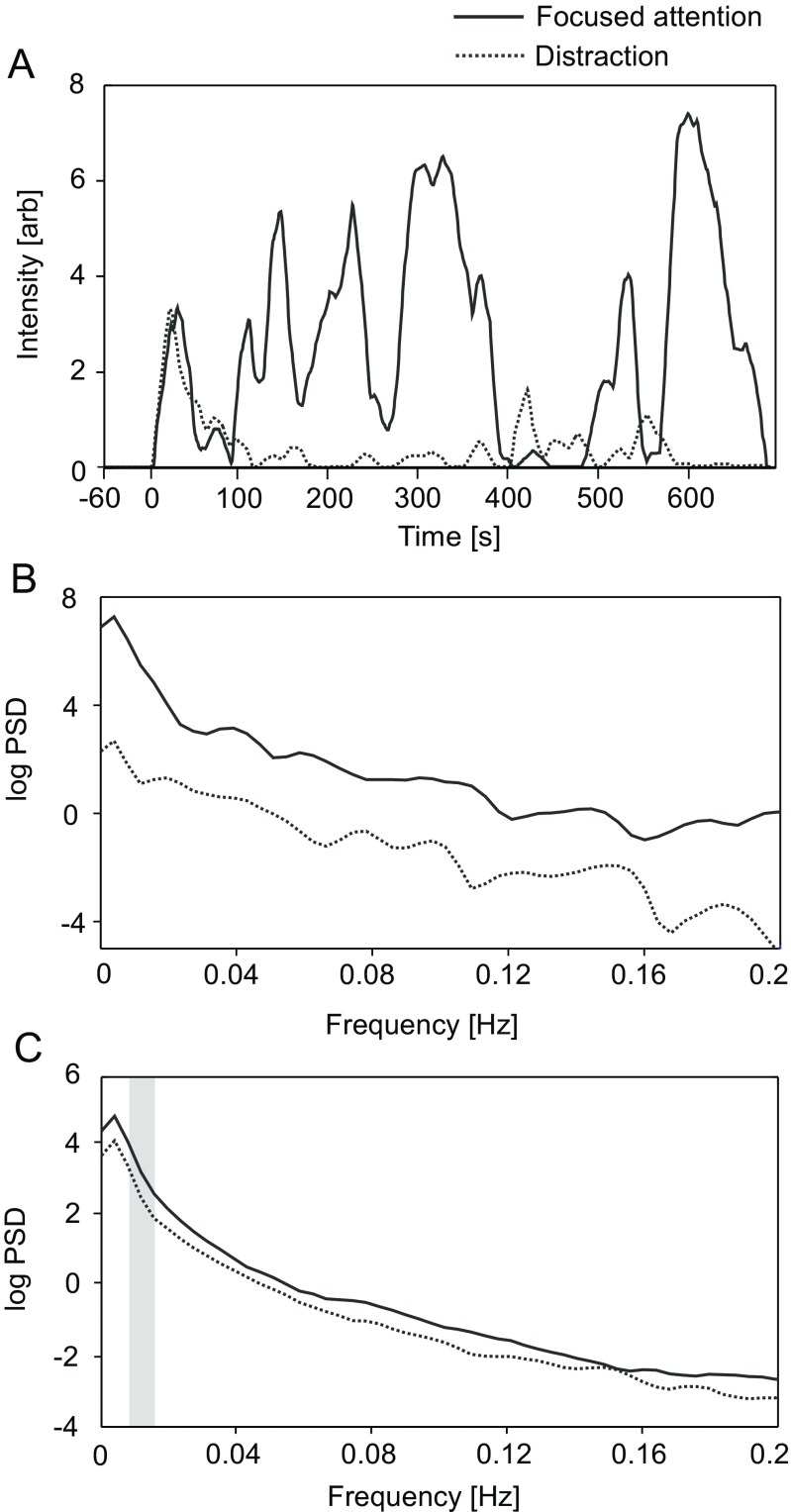
**a** Perceived odor intensity ratings for a single subject in focused attention (solid line) and distraction conditions (dashed line). **b** Log power spectral density for slow oscillations (<0.2 Hz) in same single subject for focused attention (solid) and distraction (dashed) conditions. **c** Log power spectral density for all subjects for focused attention (solid) and distraction (dashed) conditions. Gray rectangle indicates frequencies demonstrating a significant difference in relative power between conditions

To evaluate any potential spectral pattern explaining later differences in odor perception, power spectral density from 0 to 0.5 Hz (129 points) was computed for each subject and condition in the period of 120 to 660 s following onset of odor. This covers the residual exposure following the period of initial desensitization. A within-subjects *t* revealed larger log-transformed absolute spectral power for continuous intensity ratings in the focused-attention, relative to distraction, condition. This effect occurred in a frequency window ranging from 0.008 to 0.015 Hz, indicative of slow oscillatory activity with a recurring profile of between 65 and 125 s; *t*(18) = 2.47, *p* = .025 (see Fig. 3).

### Respiratory data

In the time periods 15−65 s following stimulus onset, a within-subjects *t* test revealed no significant difference in respiration rate in the focused-attention (13.39 ± 2.69) and distraction (13.64 ± 3.69) conditions; *t*(16) = −0.38, *p* = .71. Similarly, no difference was observed during the time periods 350–400 s for the focus (14.02 ± 2.77) and distraction (13.70 ± 3.23) conditions; *t*(16) = −0.34, *p* = .74, or during the period 480 to 530 s after stimulus onset for the focus (13.70 ± 2.47) and distraction (14.40 ± 2.12) conditions; *t*(16) = −1.13, *p* = .27. One-sample *t* tests to compare correlation coefficients for individual correlations between respiration rate and odor perception in the focused-attention condition did not indicate a significant relationship (*p* > .05). However, there was a borderline positive relationship between respiration rate and odor perception, t(18) = 2.03, *p* = .057, in the distracted condition. Furthermore, a paired-samples *t* test revealed a significant difference between correlation coefficients between conditions, t(18) = −2.36, *p* = .03, suggesting that respiration rate is more closely related to odor perception in distracted, relative to focused, conditions.

## Discussion

The findings demonstrate the effects of focused attention on odor desensitization, comprising a slower and linear decrease in perceived odor intensity during the first minute of odor exposure when attention was focused toward the odor, compared with a steeper, exponential decay seen in the distraction condition. Furthermore, odor desensitization was later interrupted by two recurrent periods of increased odor sensitivity highlighted by significant differences in low-frequency oscillations of subjective ratings during the focused-attention, relative to distraction, condition.

The initial, rapid decrease in odor sensitivity during the first minute of exposure was augmented in the distraction condition, which accords with previous findings indicating a significant effect of context on desensitization, particularly in the minute immediately following onset of an odor (Kobayashi et al., 2008). The pattern seen in the present study also reflects the temporal activation pattern seen in primary olfactory cortices using fMRI, manifesting a sharp decrease in activation within 1 minute of odor exposure (Sobel et al., 2000). Thus, focused attention can be seen to decrease the rate of initial desensitization seen in the period immediately following odor presentation. Previous studies indicated reduced peak intensity perception during prolonged odor perceptions in distracted conditions (Hoffmann-Hensel, Sijben, Rodriguez-Raecke, & Freiherr, 2017). While our data revealed a trend toward decreased peak odor perception in the distracted condition, this did not achieve statistical significance, which may be due to single trials necessitated by the prolonged nature of exposure in our study. The finding of recurrent periods of odor sensitivity in the focused-attention condition during the latency periods 290–350 s and 420–470 s are novel to this study. However, visual examination of data from previous research suggests that such fluctuating oscillatory profiles of odor perception during prolonged odor desensitization may also be evident in earlier studies (Dalton, 1996; Dalton et al., 1997; Kobayashi et al., 2008), although any such phenomenon has so far remained unreported. Despite using comparatively shorter odor stimuli, two brain imaging studies (Poellinger et al., 2001; Sobel et al., 2000) have also shown recurrent increases in activation of olfactory regions following the initial rapid decrease seen during desensitization. This pattern may indicate a mechanism that governs the process of switching between olfactory and other sensory processes during prolonged olfactory stimuli, similar to fluctuations of perception seen during bistable ambiguous visual stimuli (for a review, see Long & Toppino, 2004).

Our data reveal that odor desensitization demonstrate the presence of low-frequency changes in the focused-attention condition. When attention was focused toward odor, a regular pattern of odor detection appears to recur at between 1 and 2-minute intervals following the initial desensitization. This observation was confirmed by significantly greater spectral power in the 0.008−0.015 Hz range in the focused-attention, relative to distraction, condition. We conjecture that olfactory desensitization is a dynamic process, whereby attentional factors may modulate switching between odor-related percepts. Previously, attention has been shown to modulate fluctuations between perceptions of ambiguous visual stimuli (Leopold & Logothetis, 1999; Long & Toppino, 2004), or rival binocular and binaural stimuli (Blake & Logothetis, 2002; Brancucci & Tommasi, 2011; Paffen, Alais, & Verstraten, 2006). Therefore, it is likely that focused attention can also modulate the process by which the olfactory environment is continuously reevaluated during a prolonged stimulus. Additional factors could also play a relevant role in recurring perception during a dual-task process. Previous research suggests that working memory capacity could modulate orientation response to cross-modal (auditory) stimuli (Sörqvist, Nöstl, & Halin, 2012), and the role of increased cognitive load (e.g., occurring in the distraction condition) also warrants further research.

Previous studies have manipulated the context in which an odor may be perceived, and this process is likely to influence the degree of attention allocated toward an odor (Dalton, 1996). However, the primary focus of attention in such studies was always directed toward the odor. Attention for olfaction is a selective process (Keller, 2011), and the present study employed a deliberate attentional manipulation to clearly focus attention toward, or distract it away from, the odor with no manipulation of context. This type of manipulation was previously utilized to demonstrate neurophysiological effects of focused attention on odor perception using EEG (Krauel et al., 1998), and behavioral studies also demonstrated a significant effect of competing multimodal stimuli on odor perception (Spence et al., 2000; Spence, Kettenmann, et al., 2001; Spence, McGlone, et al., 2001). However, the method has, to our knowledge, never been utilized to investigate the role of attention for ongoing desensitization to prolonged stimuli. As such, the present study represents the first direct evidence that allocation of attention modulates desensitization to prolonged odor stimuli and ongoing perception of the olfactory environment, irrespective of other top-down processes. One fMRI study previously demonstrated increased cortical activations across a period of desensitization in ignore, relative to focused attention, conditions (Sabri et al., 2005). However, this research utilized detection of occurrences of deviant olfactory stimuli that differs from the present task. The contrasting findings in our study point to enhanced perception in focused attention conditions, and this difference may indicate specific mechanisms that modulate the relationship between attention and olfaction during consistent stimuli compared with novel or deviant occurrences.

The analysis of respiratory patterns indicates that the results of the present study are not attributable to differences in the rate of respiration between conditions, while the within-subjects design minimizes the influence of other physiological factors. The finding of significantly closer correlations between odor ratings and respiration rate in distracted, relative to focused, attention suggest that perception tends to follow respiration rate more closely when we are distracted. We may conjecture that this points to an increased likelihood of central factors governing the enhanced perception ratings seen in focused conditions, although further research (e.g., utilizing neuroimaging techniques) is needed to confirm this.

The relatively small number of participants should be considered as a limitation of the present design, and further research is needed to replicate and expand on the present findings. Likewise, the odor utilized in the present study exhibited little or no trigeminal profile, but future studies should compare the role of trigeminal, relative to purely olfactory, contributions for recurring odor perception during desensitization. For curve fitting, we opted for a group-level method based upon classical analyses of human olfactory stimulus–response curves (Chastrette, Thomas-Danguin, & Rallet, 1998). An alternative, even preferable, method would have been to evaluate individual response curves prior to group-level tests (Sinding et al., 2017). However, this method was not selected due to the reduced signal-to-noise inherent in single prolonged exposures which is a limitation of the current study design.

In conclusion, our findings show modulation of odor desensitization by focus of attention for the first time, exemplifying the importance of top-down control of odor perception. Distraction appears to increase the rate at which desensitization occurs in the initial stages of odor exposure, but also reduces ongoing recurrent sensitivity to odor. Further research is required to investigate the precise time course of this latter phenomenon in particular, for example, to evaluate whether the effect is stable across populations, or odors of differing hedonics or intensities.

## Electronic supplementary material

Supplementary Fig. S1 (PDF 78 kb)
