# Supplementary material for: Attentional modulation of desensitization to odor
Source: Atten Percept Psychophys. 2018 May 22;80(5):1064–71. doi: 10.3758/s13414-018-1539-2 (PMC6061002; doi:10.3758/s13414-018-1539-2)
Supplement: Supplementary file 1 — (PDF 78 kb) [file 13414_2018_1539_MOESM1_ESM.pdf]

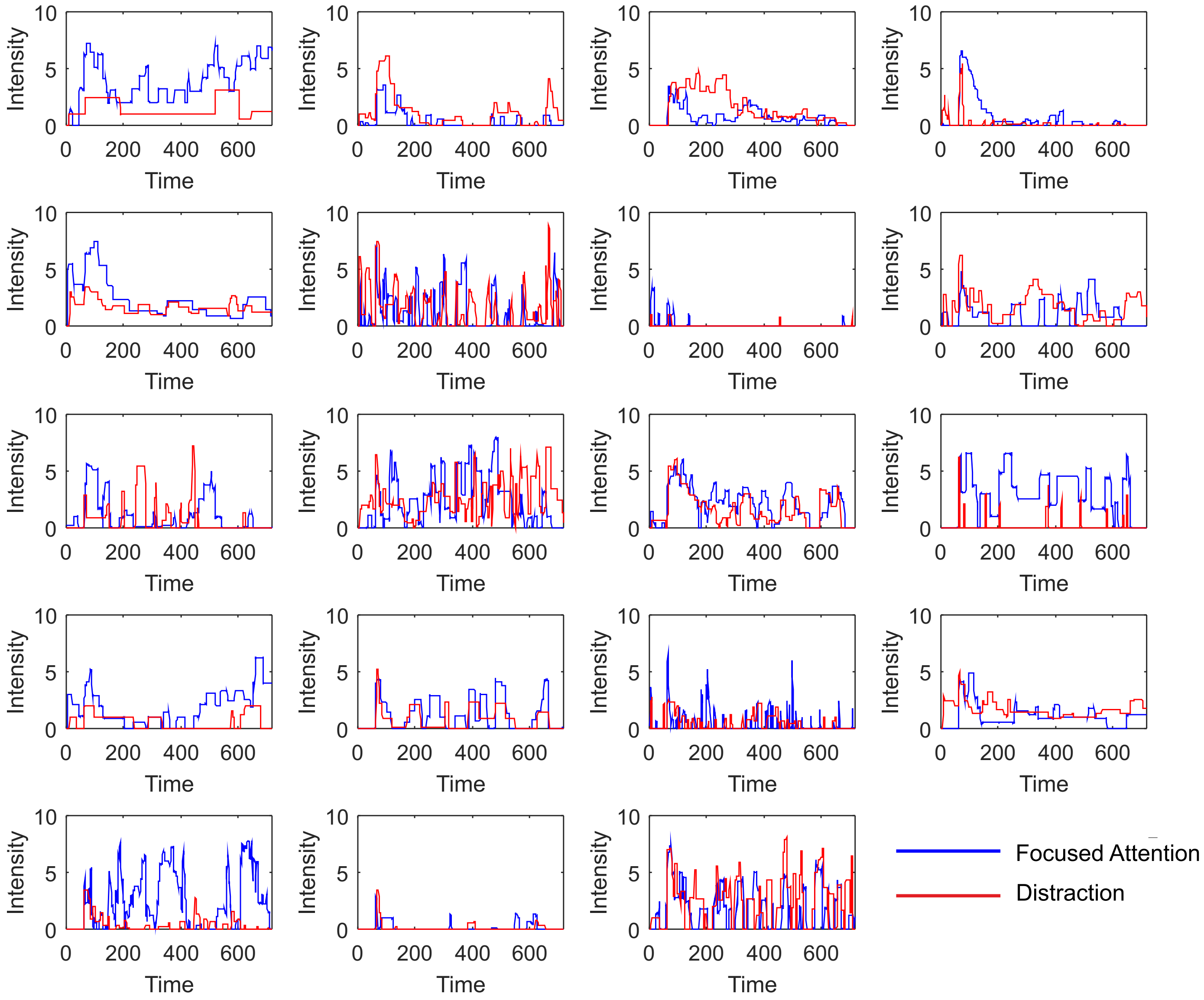

Supplementary Figure 1. Individual perceived intensity ratings and desensitization curves. The blue line indicates intensity ratings (arbitrary units) in each individual subject for all 720 timepoints in the focused attention condition; red colour indicates the intensity ratings during the distraction condition.
